# A Proposed Method for Estimating Refractive Error in Primary School Children

**DOI:** 10.7759/cureus.34554

**Published:** 2023-02-02

**Authors:** Feride Tuncer Orhan, Haluk Huseyin Gursoy

**Affiliations:** 1 Ophthalmology, Eskisehir City Hospital, Eskisehir, TUR; 2 Ophthalmology, Eskisehir Osmangazi University, Eskisehir, TUR

**Keywords:** pediatric myopia, emmetropization, anterior chamber depth, keratometry, hypermetropia, spherical equivalent, refractive errors, myopia progression, biometry, axial length of orbit

## Abstract

Background

This study aimed to evaluate consecutive measurements of biometric parameters, age, and refraction in a cohort of Turkish primary school-age children and to assess the correlation between biometric changes and refraction.

Methodology

The study population was seven and 12-year-old children (n = 197). The retrieved data consisted of three consecutive measurements with a one-year interval for each subject. Data from one eye (right) were used. Age, gender, body mass index, spherical equivalent (SE), axial length (AL), anterior chamber depth (ACD), central corneal thickness (CCT), keratometry (K), and lens thickness (LT) were analyzed. The onset and final data were retrieved from the database in 2013 and 2016, respectively. Statistically, logistic and Cox regression models of all parameters were analyzed, and the significance level was set at 5%.

Results

The median of the onset and final SE values were -0.00 D (0.00-0.00) and 0.50 D (0.19-1.00), respectively. The onset AL (hazard ratio (HR) = 5.82, 95% confidence interval (CI) = 3.45-9.76, β = 1.76, p < 0.001), K_mean _(HR = 2.28, 95% CI = 1.67-3.11, β = 0.82, p < 0.001), and age (HR = 0.77, 95% CI = 0.59-0.99, β = -0.26, p = 0.046) were correlated with myopia progression. To calculate the estimated SE, the onset data were included in the logistic regression model. The onset SE (β = 0.916, p < 0.001), AL (β = -0.451, p < 0.001), ACD (β = 0.430, p = 0.005), and K (β = -0.172, p < 0.001) were correlated with the mean final SE. An equation was generated using the regression model analysis.

Conclusions

The onset parameters of SE, AL, ACD, and K were confirmed to correlate with the final SE values in the proposed model. To confirm the use of the refractive calculator, a cross-validation analysis is needed to estimate three-year subsequent refractive error among seven and 12-year-old children.

## Introduction

Uncorrected refractive errors, especially myopia, are the leading disorders that cause vision loss globally [1]. Recently, it has been reported that over 157 million people suffer from vision loss related to mid or severe refractive disorders globally [1]. The estimated cost of this burden to the global economy has been reported to be over US $269 billion annually [2]. Accordingly, refractive defects present a major public health problem in the world.

The most common type of refractive error is hypermetropia in the postnatal term [3]. The postnatal hyperopic state regresses over time due to physiological emmetropization mechanisms associated with alterations in the axial length of the eyeball, lens, and corneal refractive power. If the emmetropization mechanism fails or is delayed during this period due to environmental or hereditary reasons, refractive errors occur [4]. Although underlying risk factors and onset mechanisms are known, the underlying mechanisms of refractive errors are not fully understood yet.

Epidemiological indicators have shown that there has been a significant increase in the prevalence of myopia in the last decade [1]. Clinically, early diagnosis of initial myopia and progression is crucial to control this refractive error. Early myopia diagnosis is necessary to initiate a myopia control treatment and reduce the complications associated with myopia. Eventually, uncorrected myopia may have a risk of complications related to the detachment or neovascularization of the retina, early cataracts, and glaucoma [5]. These complications are not only related to the high socioeconomic treatment costs but also may result in irreversible conditions for patients, such as blindness.

Clinical studies are essential to analyze the refractive errors and ocular biometric changes of the population in the onset age range of myopia [6]. Such information is also necessary to understand the progressive nature of refractive errors. Rozema et al. have reported that monitoring the progression of ocular biometric parameters in school-age children up to adolescence is an ideal method for interpreting biometric changes related to the initiation of myopia [7]. However, the above-mentioned epidemiologic data of primary school children are not available for the Turkish population in the current literature.

Wolffsohn et al. have reported that potential myopia calculators can be beneficial tools reflecting the average potential outcomes based on research data [8]. The authors have emphasized that the data should be collected based on carefully selected cases examined for two and five years only. Very recently, myopia progression estimated by the Brien Holden Vision Institute Myopia Calculator with cycloplegic measures in children in Hong Kong wearing single-vision distance spectacles over a one and two-year period was compared [9]. Further, it has been reported that such optic calculators can act as a guide to estimate the risk of developing myopia. Possibly, such optic calculators can be employed as a guide to estimate the risk of developing myopia [10].

To seek the potential correlations with spherical refraction, this study aimed to evaluate consecutive measurements of biometric parameters, age, and refraction in a cohort of Turkish primary school-age children and to assess the correlation between biometric changes and refraction.

## Materials and methods

Study population and inclusion criteria

In this study, patient records were obtained from the database of the Department of Ophthalmology, Eskisehir Osmangazi University, Eskisehir, Turkey. The retrieved data consisted of three consecutive measurements with a one-year interval for each subject. We retrospectively reviewed records of patients who underwent full ocular examinations between January 2013 and January 2016. Finally, 197 patients were included in the study. The study was performed in accordance with the Helsinki Declaration of 1975, as revised in 2000. The a priori logistic regression was computed using G*Power version 3.1 (Düsseldorf, Germany). The input parameters were as follows: odds ratio (OR): 2.34, power (1-β): 80%, R²: 0.04, X distribution: binomial, X parm π: 0.5, and α= 0.05. The required total sample size was calculated as 155 (critical z output: 1.65, actual power: 0.80). The assessed final sample size was higher than the calculated sample size.

Children aged 7-12 years whose records were available in the Pediatric Ophthalmology Unit of the Department of Ophthalmology were consecutively selected for routine ocular examinations between January 2013 and January 2016. The data of three measurements of children aged 7-12 years who were examined at least once a year for three years using both cycloplegic auto-refractometry (RM-A7000B; Topcon Medical Systems, Inc., Oakland, NJ) and optical biometry (Lenstar LS900; Haag-Streit Diagnostics AG, Koeniz, Switzerland) were included as the cohort data for this study. Data from one eye (right) were used.

The main inclusion criteria for subjects were no additional ocular problems except refractive errors. Further, subjects who had no refractive error at the start of the three years were also included. Children with ptosis, pterygium, dry eye, corneal disease, cataracts, retinal disease, strabismus, contact lens usage history, previous ophthalmic surgery in any eye, or uncooperative children were not included in the study. The common inclusion criteria were children with no systemically compromised conditions and those having fully available records with auto-refractometry and optical biometry measurements in all observation periods.

Ocular examination procedures

All examinations were performed by ophthalmologists with two to four years of clinical experience using both instruments registered in the unit following the manufacturers’ instructions. Spherical equivalents (SEs) were calculated by adding the sum of the sphere power with half of the cylinder power obtained by the Topcon RM-A7000B instrument. Myopia was defined as an SE of ≤-0.75 D, hyperopia was defined as an SE of ≥+0.75 D, and emmetropia was defined as an SE between +0.50 and -0.50 D. Axial length (AL), anterior chamber depth (ACD), central corneal thickness (CCT), keratometry (K), and lens thickness (LT) were measured using the Lenstar LS 900 instrument. K_mean_ shows the arithmetic mean of K1 and K2 values.

Baseline parameters of age, gender, height, body mass index (BMI), SEs, AL, ACD, CCT, K_mean_, and LT were analyzed and compared annually including the final data. The baseline data was from 2013 and the final data was from 2015. In this study, the onset datum was defined as the corresponding parameter when children came for the first time to the hospital. Accordingly, the onset age was defined as the age when children came for the first time to the hospital.

Statistical analysis

Statistical analyses were performed using SPSS version 21.0 (IBM Corp., Armonk, NY, USA). To determine the differences between variables (age or refractive group myopic versus non-myopic groups) per difference in time (onset and three years after) statistical analyses were performed. The normality of data was examined using the Shapiro-Wilk normality test. For normally distributed data, the independent t-test and one-way analysis of variance (ANOVA) test were performed for comparisons. For non-normally distributed data, the Mann-Whitney U test and the Kruskal-Wallis test were performed for comparisons. Logistic regression and Cox regression models of parameters were analyzed to identify correlations of risk factors. Statistical significance was assumed at p-values <0.05.

## Results

Biometric measurements

A total of 671 records were assessed. Of the 671 subjects, 474 were excluded from the study due to not having consecutive follow-up visits or not meeting the inclusion criteria. In total, 197 eyes were included in the study according to the inclusion criteria. Of the subjects, 48.7% (n = 96) were females and 51.3% (n = 101) were males. At the onset, the age of the study cohort was 9.58 ± 1.56 years. The prevalence of onset refractive errors of emmetropia, hypermetropia, and myopia was 81.7% (n = 161), 13.2% (n = 26), and 5.1% (n = 10), respectively. The descriptive data of onset and final SE values are presented in Table 1. The median of the onset and the final SE values were 0.0 D (0.00-0.00), and 0.50 D (0.19-1.00), respectively.

**Table 1:** The mean and standard deviations of onset and final biometric measurements by age. for normally distributed data, independent t-tests and one-way ANOVA tests were performed for comparisons. For non-normally distributed data, Mann-Whitney U and Kruskal-Wallis tests were performed for comparisons. The superscript letters represent the findings of the pairwise comparisons between groups. These letters are placed at the end of the means in each column for each biometric parameter where the significance is seen. The superscript letters mean significant differences (p < 0.05). *: Significant biometric parameters by age (p < 0.05). SE = mean spherical equivalent; AL = axial length; ACD = anterior chamber depth; LT = lens thickness; CCT = central corneal thickness; K_mean_ = mean keratometry

Biometric parameter	Age	n	Retrospective data		
			Initial	Final	Change over
SE	7	26	0.5 (0–1.25)	0.93 (0.25–1.25)	0,19 (-0.50–0.53)
	8	25	0 (0–0)	0.50 (0–1.13)	0.25 (-0.50–0.81)
	9	38	0.25 (0–0.5)	0.31 (-0.50–1.16)	0.31 (-0.50–1.16)
	10	40	0 (0–0)	0.50 (-0.06–1)	0.50 (0.06–1)
	11	34	0 (0–0)	0.38 (-0.06–1)	0.31 (-0.25–1)
	12	34	0.50 (-0.75–1)	0.25 (-1.41–0.78)	-0.25 (-0.53–0)
Total		197	0 (0–0)	0.50 (0.19–1)	0.25 (-0.37–0.75)
AL	7	26	22.57 ± 0.81 ^a^	23.11 ± 0.88	0.54 ± 0.23^ a^
	8	25	22.82 ± 0.83 ^ab^	23.30 ± 1.01	0.48 ± 0.73 ^ab^
	9	38	23.14 ± 0.81 ^ab^	23.45 ± 0.91	0.30 ± 0.22 ^bc^
	10	40	23.03 ± 0.66 ^ab^	23.26 ± 0.68	0.23 ± 0.16 ^c^
	11	34	23.29 ± 0.81^b^	23.42 ± 0.85	0.13 ± 0.10 ^c ^
	12	34	23.45 ± 1.03 ^b^	23.68 ± 1.06	0.23 ± 0.17 ^c ^
Total		197	23.08 ± 0.86	23.38 ± 0.90	0.30 ± 0.33
ACD	7	26	2.89 ± 0.26 ^a^	3.14 ± 0.27	0.25 ± 0.14^ a^
	8	25	3.02 ± 0.28^ a^	3.17 ± 0.30	0.16 ± 0.20 ^ab^
	9	38	3.12 ± 0.21^ ab^	3.21 ± 0.20	0.10 ± 0.05^ bc^
	10	40	3.14 ± 0.22 ^ab^	3.25 ± 0.23	0.12 ± 0.09^ bc^
	11	34	3.19 ± 0.28 ^ab^	3.26 ± 0.30	0.05 ± 0.07^ bc^
	12	34	3.27 ± 0.30 ^b^	3.29 ± 0.30	0.05 ± 0.06^ c^
Total		197	3.12 ± 0.28	3.23 ± 0.27	0.12 ± 0.12
LT	7	26	3.52 ± 0.20^ a^	3.35 ± 0.15	-0.17 ± 0.17^ a^
	8	25	3.45 ± 0.24 ^ab^	3.29 ± 0.23	-0.17 ± 0.31^ a^
	9	38	3.36 ± 0.18 ^ab^	3.33 ± 0.18	-0.04 ± 0.09^ ab^
	10	40	3.41 ± 0.17 ^ab^	3.38 ± 0.17	-0.04 ± 0.09^ ab^
	11	34	3.36 ± 0.16 ^ab^	3.47 ± 0.44	0.10± 0.47^ b^
	12	34	3.31 ± 0.34 ^b^	3.30 ± 0.21	-0.05 ± 0.35^ ab^
Total		197	3.40 ± 0.23	3.36 ± 0.26	-0.05 ± 0.29
CCT	7	26	547.50 ± 34.98	549.15 ± 36.01	1.65 ± 0.60
	8	25	531.92 ± 38.90	528.80 ± 43.57	-3.12 ± 16.03
	9	38	554.71 ± 31.60	554.21 ± 33.13	-0.50 ± 4.40
	10	40	540.43 ± 31.56	541.85 ± 33.69	1.42 ± 8.48
	11	34	551.04 ± 34.50	550.0 ± 34.71	-1.04 ± 4.67
	12	34	552.56 ± 29.52	550.12 ± 30.37	-2.44 ± 4.51
Total		197	546.96 ± 33.66	546.38 ±35.36	-0.59 ± 7.88
K_mean_	7	26	43.18 ± 1.86	43.21 ± 1.81	0.02 ± 0.14
	8	25	43.49 ± 1.19	43.41 ± 1.18	-0.08 ± 0.43
	9	38	43.69 ± 1.78	43.68 ± 1.80	-0.01 ± 0.13
	10	40	43.67 ± 1.48	43.64 ± 1.43	-0.03 ± 0.23
	11	34	43.15 ± 1.39	43.11 ± 1.38	-0.03 ± 0.15
	12	34	43.53 ± 1.53	43.62 ± 1.56	0.09 ± 0.15
Total		197	43.47 ± 1.56	43.46 ±1.55	-0.01 ± 0.22

The mean and standard deviations of onset and final biometric measurements by age are also presented in Table 1. There were significant differences between onset age and biometric parameters. Accordingly, the increased onset age was due to a significant increase in AL (p = 0.001) and ACD (p < 0.001) while it was due to a significant decrease in LT (p = 0.007). The CCT and K_mean_ progression by onset age were not significant (p = 0.074 and p = 0.579, respectively). In comparisons of the biometric measurements by consecutive follow-up visits, there were significant differences in AL (p < 0.001), ACD (p < 0.001), and LT (p = 0.09). In younger onset age, the progression rate of AL and ACD were higher than older onset age whereas the regression rate of LT was lower. In final visits, these significant differences between biometric measurements and final age were lost (p > 0.05).

Progression of refractive error

The mean and SDs of the onset and final biometric measurements by refractive error type are given in Table 2. It was noted that the prevalence of myopia increased from 5.1% (n = 10) to 16.2% (n = 32) within the observation period. However, the prevalence of hypermetropia increased from 13.2% (n = 26) to 41.1% (n = 81).

**Table 2:** The mean and standard deviations of the onset and final biometric measurements by refractive error type. AL = axial length; ACD = anterior chamber depth; LT = lens thickness; CCT = central corneal thickness; K_mean_ = mean keratometry

Biometric parameter	Refractive error type	Initial		Final		Change over	
		Mean ± SD	P-value	Mean ± SD	P-value	Mean ± SD	P-value
AL	Myopia	24.24 ± 1.15	<0.001	24.27 ± 0.86	<0.001	0.53 ± 0.31	<0.001
	Non-myopia	23.02 ± 0.80		23.21 ± 0.81		0.26 ± 0.32	
ACD	Myopia	3.38 ± 0.28	0.002	3.36 ± 0.22	0.002	0.10 ±0.09	0.520
	Non-myopia	3.10 ± 0.27		3.20 ± 0.27		0.12 ± 0.13	
LT	Myopia	3.21 ± 0.12	0.008	3.30 ± 0.16	0.201	-0.04 ± 0.10	0.855
	Non-myopia	3.41 ± 0.23		3.37 ± 0.27		-0.05 ± 0.31	
CCT	Myopia	563.89 ± 33.05	0.103	547.25 ± 32.84	0.879	-1.03 ± 5.59	0.729
	Non-myopia	546.06 ± 33.53		546.21 ± 35.92		-0.50 ± 8.27	
K_mean_	Myopia	43.80 ± 1.45	0.488	43.93 ± 1.58	0.064	0.07 ± 0.14	0.034
	Non-myopia	43.45 ± 1.56		43.37 ± 1.53		-0.02 ± 0.23	

In the comparison of the onset biometric measurements between myopic and non-myopic children, AL was longer (p < 0.001), ACD was deeper (p = 0.002), and LT was thinner (p = 0.008) in myopic subjects. In myopic patients, the mean SE decreased by 1.55 D (±1.31) in three years (p < 0.001), while the mean AL increased by 0.53 mm (0.31) (p < 0.001). In non-myopic patients, the mean SE increased by 0.47 D (0.78) and the mean AL was 0.26 mm (0.32) in three years (p < 0.001).

Factors associating the final SE of 32 children with myopia progression were evaluated by Cox regression analysis including the onset biometric parameters given in Table 3. SE was significantly correlated by age (hazard ratio (HR) = 0.77, 95% confidence interval (CI) 0.59-0.99, β = -0.26, p = 0.046), AL (HR = 5.82, 95% CI = 3.45-9.76, β = 1.76, p < 0.001), and K_mean_ (HR = 2.28, 95% CI = 1.67-3.11, β = 0.82, p < 0.001).

**Table 3:** Cox regression analysis of factors correlating the mean spherical equivalent values on myopia. OD = odds ratio; HR = hazard ratio; SE = mean spherical equivalent; AL = axial length; ACD = anterior chamber depth; LT = lens thickness; CCT = central corneal thickness; K_mean_ = mean keratometry; CI = confidencel interval

Parameters	P-value	β	HR	OD (95% CI)	
				Minimum	Maximum
Gender	0.70	-0.16	0.85	0.38	1.93
Age	0.07	-0.36	0.70	0.47	1.03
Height	0.50	0.00	1.00	0.99	1.01
Weight	0.32	0.03	1.04	0.97	1.10
AL	0.00	2.01	7.45	3.70	15.00
ACD	0.24	-1.24	0.29	0.04	2.30
LT	0.80	0.28	1.32	0.16	10.94
CCT	0.84	-0.00	1.0	0.99	1.01
K_mean_	0.00	0.87	2.40	1.69	3.41
Age	0.046	-0.26	0.77	0.59	0.99
AL	<0.001	1.76	5.82	3.45	9.76
K_mean_	<0.001	0.82	2.28	1.67	3.11

Calculation of estimated refractive error

Factors correlating the average SE at the end of three years were evaluated by logistic regression. Logistic regression analysis of factors correlating mean SE is given in Table 4. For this purpose, the onset biometric and demographic (age, gender, weight, and height) data were included in the logistic regression model. The onset data of SE (β = 0.916, p < 0.001), AL (β = -0.451, p < 0.001), ACD (β = 0.430, p = 0.005), and K_mean_ (β = -0.172, p < 0.001) were found to be significantly associated with the mean SE at the final data. However, demographic onset data were not significantly correlated with the mean SE in the final data (p > 0.05). The coefficient of determination (R^2^) of regression was set at 0.761 for these variables. To calculate the estimated SE after three years, the equation was established using the logistic model.

**Table 4:** Logistic regression analysis of factors correlating the mean spherical equivalent values. SE = mean spherical equivalent; AL = axial length; ACD = anterior chamber depth; LT = lens thickness; CCT = central corneal thickness; K_mean_ = mean keratometry

Independent variable	Regression coefficient	P-value
Constant	21.83	<0.001
SE	0.82	<0.001
AL	-0.51	0.031
ACD	0.37	0.017
LT	-0.21	0.170
CTT	0.00	0.803
Height	0.01	0.050
Weight	-0.01	0.061
K_mean_	-0.20	<0.001
Gender	0.14	0.029
Age	0.01	0.786
Constant	16.46	<0.001
SE	0.916	<0.001
AL	-0.451	<0.001
ACD	0.430	0.005
K_mean_	-0.172	<0.001

SE_3_ = [16.46 + (0.916 × SE) + (0.430 × ACD)] - [(0.451 × AL) + (0.172 × K_mean_)]

SE_3_ shows the estimated SE after three years, SE shows the onset spherical equivalent, ACD shows the onset anterior chamber depth, AL shows the onset axial length, and K_mean _shows the onset arithmetic mean of keratometry. The unit of SE is in diopters and the units of ACD, AL, and are in millimeters. Lastly, reliability and validity checks of the estimation model were conducted deductively using our retrospective onset dataset created by MS Excel.

## Discussion

We analyzed the refractive errors and ocular biometric changes over time in a Turkish population at the onset age range of myopia over time. Regarding the onset age of the cohort, there were significant differences between onset biometric parameters and age. Accordingly, the increased onset age was due to a significant increase in AL (p = 0.001) and ACD (p < 0.001) while it was due to a significant decrease in LT (p = 0.007). Regarding consecutive follow-ups, there were significant differences in AL (p < 0.001), ACD (p < 0.001), and LT (p = 0.009). In younger onset age, the progression rates of AL and ACD were higher than in older onset age whereas the regression rate of LT was lower. Interestingly, these significant differences between biometric measurements and final age were lost at the final visits (p > 0.05). Only myopia prevalence was increased in observation time periods. Regarding the Cox regression results, myopic SE was significantly correlated by age (HR = 0.77, 95% CI = 0.59-0.99, β = -0.26, p = 0.046), AL (HR = 5.82, 95% CI = 3.45-9.76, β = 1.76, p < 0.001), and K_mean_ (HR = 2.28, 95% CI = 1.67-3.11, β = 0.82, p < 0.001). In the cohort study, factors correlating the average SE at the end of three years were evaluated by logistic regression. The onset data of SE (β = 0.916, p < 0.001), AL (β = -0.451, p < 0.001), ACD (β = 0.430, p = 0.005), and K_mean_ (β = -0.172, p < 0.001) were found to be significantly associated with the mean SE at the final data and, therefore, the null hypothesis was rejected.

As an output of the statistical analyses of this study, an equation was proposed using the logistic regression model for calculating the estimated SE after three years. The proposed equation uses the onset SE, ACD, AL, and K_mean_ parameters as inputs to calculate the estimated SE after three years. Regarding the proposed equation, the output is positively (increasingly) impacted by the onset of SE and ACD, while it is negatively (tends to decrease) impacted by the onset of AL and K_mean_.

Physiologically, biometric parameters such as AL, ACD, LT, and corneal power could affect the steadily refractive condition of the eye [11]. Correspondingly, the onset of myopia or its progression could be seen during the enlargement of AL, which could not be tolerated naturally [4]. More specifically, 5-15-year-old children are considered as the onset age of myopia [12,13]. It has been reported that observing the changeover of optic biometric values in school-age children up to adolescence is an ideal method for interpreting the initiation of myopia [7]. Besides, it has been reported that the increasing onset age of young patients could be one of the significant variables correlating clinical outcomes in a previous report [12]. Hence, the proposed equation in this study should be considered specific for children between the ages of seven and 12. When it is employed at younger ages or older ages than the cohort, the progression cannot be estimated usingthe proposed calculator due to unique developmental mechanisms [14,15].

Potential myopia calculators could be beneficial tools to reflect the average potential outcome [8]. In addition, it has been reported that such a tool built on the data should be collected from cases examined for between two and five years [8]. In agreement with the previous report, the underlying data of the proposed calculator were collected from cases examined for three years. For the first time, logistic regression was employed to propose an SE calculator in 7-12-year-old children. Regarding the proposed calculator, associated biometric variables were identified as the onset values of SE, ACD, AL, and K_mean_. The input parameters consisted of the department database records collected from registered instruments (Topcon RM-A7000B and Lenstar LS900). Hence, data variability might be possible when the proposed calculator is validated with different optical biometry instruments due to incompatibility. The rationale for this phenomenon, the low agreement status in interchangeably used biometry instruments has been also reported in a previous study [16].

The environmental factors regarding the onset of myopia in children are the outcomes of close-up physical activities [17,18]. However, this retrospective study cannot standardize individual environmental factors. The retrospective design of this study was considered a limitation of the study. As the nature of a retrospective study, selection bias could occur unintentionally among the patients. The present study described the calculation of estimated refractive error after three years using baseline biometric values in primary school children. Only patients with consecutive measurements for three years were included. Consequently, we found that the three-year estimation of the refractive error correlated regularly with initial parameters in 7-12-year-old children. Regarding the proposed logistic model, it could be used for patients with developing or progressing myopia for scheduling follow-ups and treatment planning purposes. Moreover, in patients with fewer refractive errors, follow-ups could be made less often. With this motivation, further prospective studies are needed to validate the proposed logistic regression equation in follow-up patients.

In this study, the issue was assumed as a linear function to estimate myopia based on regression models. The changes in myopia could behave as a logarithmic or polynomial function non-linearly. We accept this as a limitation of the proposed calculator.

In this cohort study, as data from Eskisehir city and its surroundings were analyzed, the outcomes might not reflect the general Turkish population or other countries. However, the primary purpose of this study was not to conduct a demographic study.

It has been reported normative values for AL can be used to monitor eye growth in European children at both six and nine years of age in a previous study [19]. Similarly, Sanz Diez et al. [20] have reported a clinical model to predict myopia development based on the creation of percentile curves of AL in school-aged children from Wuhan in central China. Very recently, Truckenbrod et al. [21] have reported correlation curves for AL by SE, age, or gender in German children aged 2-18 years. In addition, the authors have concluded that the percentile curves of AL can be used as a predictive measure for the likelihood of developing as well as the progression of myopia [21]. In agreement with the previous reports, AL was one of the biometric variables in our generated model in the present study. Sanz Diez et al. [20] have used successive measurements of 226 children to verify the predictive power of AL growth percentile curves. In agreement with the previous report, data validation was performed on the dataset of included children (n = 197, 100%) in this study. The equation or the calculation model was first generated in this study. Although a small sample size was used compared to previous reports, the model was validated with our dataset.

## Conclusions

Within the limitations of this study, it can be concluded that the onset parameters of SE, AL, ACD, and K were confirmed to correlate with the final SE values in the proposed model. To confirm the use of a refractive calculator, a cross-validation analysis is necessary to estimate three-year subsequent refractive errors among 7-12-year-old children.
